# The COVID-19 pandemic in children and young people during 2022–24: what new did we learn?

**DOI:** 10.7189/jogh.15.01002

**Published:** 2025-04-02

**Authors:** Igor Rudan, Steven Kerr, Colin R Simpson, Amanj Kurdi, Davies Adeloye, Chris Robertson, Aziz Sheikh, EAVE II Collaboration

**Affiliations:** 1 Usher Institute, The University of Edinburgh, Edinburgh, UK; 2 Nuffield Department of Primary Care Health Sciences, Oxford University, UK; 3 School of Health, Wellington Faculty of Health, Victoria University of Wellington, Wellington, New Zealand; 4 Strathclyde Institute of Pharmacy and Biomedical Sciences, Strathclyde University, Glasgow, UK; 5 College of Pharmacy, Hawler Medical University, Erbil, Kurdistan Region, Iraq; 6 College of Pharmacy, Al-Kitab University, Kirkuk, Iraq; 7 Department of Public Health Pharmacy and Management, School of Pharmacy, Sefako Makgatho Health Sciences University, Pretoria, South Africa; 8 Teesside University, Middlesborough, UK; 9 Department of Mathematics and Statistics, University of Strathclyde, Glasgow, UK; 10 Public Health Scotland, Glasgow, UK

## Abstract

The research conducted between 2022 and 2024 has advanced our understanding of COVID-19 in children and young people (CYP), particularly with the emergence of the Omicron variant and its subvariants. The findings have reinforced that, while Omicron infections are often milder compared to earlier variants, the overall seroprevalence of SARS-CoV-2 in children has increased, with notable regional and demographic disparities. COVID-19-related hospitalisation rates in children rose during Omicron waves, especially among infants, unvaccinated individuals, and CYP at higher risk, *i.e.* with comorbidities such as obesity, diabetes, and neurological or cardiac conditions. Despite this, severe disease and mortality in children remained very low. The observed increases in type 1 diabetes incidence and multisystem inflammatory syndrome in children (MIS-C) have also highlighted the broader systemic effects of SARS-CoV-2 in paediatric populations. Evidence has underscored the protective effect of vaccination in preventing severe disease and MIS-C and vaccine safety, emphasising the need for targeted immunisation strategies, particularly among children who may be at higher risk. Studies have also estimated that a significant proportion of children experienced persistent post-COVID-19 infection symptoms such as fatigue, mood disturbances, sleep disorders, and respiratory difficulties, but the reported prevalence varied widely, from as low as 1.6% to as high as 70%, due to differences in study methodologies, case definitions, and populations studied. Standardised definitions and measurement tools, such as those developed through international consensus processes, are required to improve diagnosis, treatment, and research into this persisting condition. Ethnic disparities in vaccine uptake persist, implying that vaccine hesitancy and accessibility, alongside approaches to countering disinformation, are important areas for future research.

In our previous two editorials, published in December 2021, we reviewed early research evidence into SARS-CoV-2 infection and the COVID-19 pandemic in children and young people (CYP) [1,2]. In 2020 and 2021, the research in many countries primarily focussed on clinical presentation, transmission patterns, viral load, diagnostic approaches, and treatment strategies [1]. In CYP, diagnostic methods to confirm current or past SARS-CoV-2 infection have included reverse transcription polymerase chain (RT-PCR) testing, rapid antigen tests, and serology. Treatment is mainly supportive, with antivirals and glucocorticoids reserved for severe cases [3,4]. There was also the complex question of the safety and effectiveness of vaccination in this age group, with a need for robust evidence from clinical trials and 'real-world' observational studies to fill the knowledge gap [2]. In this editorial, we reflect on the important new knowledge related to COVID-19 in CYP that was added to the world’s literature in 2022–24.

## NEW VARIANT OF SARS-COV-2 – OMICRON

The Omicron variant (B.1.1.529) was first reported following a substantial increase in COVID-19 cases from mid-November 2021, starting in Tshwane District, Gauteng province, South Africa. An early seroepidemiological survey in Gauteng determined the seroprevalence which ranged from 56.2% among children <12 years to 79.7% among adults >50 years. Vaccinated participants were more likely to be seropositive than unvaccinated ones (93.1% *vs*. 68.4%). Epidemiological data showed that the incidence of SARS-CoV-2 infection increased and then declined more rapidly during the fourth, Omicron wave than it had during the three previous ones [5]. This new wave coincided with an increase in paediatric COVID-19-associated admissions to hospital. There was uncertainty about whether this was due to increased transmission and infection rates or increased severity due to the new variant, prompting new research on the effects of the Omicron variant on children and adolescents [6].

## NATURAL HISTORY OF OMICRON INFECTIONS.

A meta-analysis of eight studies covering 7640 Omicron variant-positive individuals identified that the pooled percentage of asymptomatic infections was 32.4%. Asymptomatic infections were more frequent in the populations of low- and middle-income countries, those with vaccine coverage ≥80%, among persons with a travel history, community infection spread, and those with a median age <20 years. In other groups, symptoms of varying degrees of severity may be noticed, but they are generally milder than for the previous variants [7].

## CHANGES IN GLOBAL SEROPREVALENCE OF SARS-COV-2

A review of 965 distinct seroprevalence studies sampling 5 346 069 participants between January 2020 and April 2022 estimated that, by September 2021, global SARS-CoV-2 seroprevalence from infection or vaccination was 59.2%. Overall, seroprevalence rose steeply in 2021 due to infection in some regions (*e.g.* to 86.7% in Africa by December 2021) and vaccination and infection in others (*e.g.* to 95.9% in December 2021 in European high-income countries). After the emergence of Omicron in March 2022, infection-induced seroprevalence rose to 47.9% in high-income countries of Europe and 33.7% in high-income countries of the Americas. Children from 0–9 years and adults aged ≥60 were less likely to be seropositive than adults aged 20–29 years [8].

Since the emergence of the immune-evasive SARS-CoV-2 Omicron variant and its subvariants, the higher hospitalisation rates of CYP aged 0–19 years highlighted a need for further investigation of SARS-CoV-2 seroepidemiology in children. Pooled seroprevalences were estimated according to World Health Organization (WHO) regions using random-effects meta-analyses. They varied from 7.3% in the first wave of the COVID-19 pandemic to 37.6% in the fifth wave and 56.6% in the sixth wave, when Omicron was dominant. The highest seroprevalences in different pandemic waves were estimated for South-East Asia and African regions, while the lowest was estimated for the Western Pacific region. Seroprevalence estimates were higher in older children, in those living in low- and middle-income countries or regions, and in those of minority ethnic backgrounds. By the end of 2021 and before the Omicron wave, around 50–70% of children globally were still susceptible to SARS-CoV-2 infection [9].

## SEVERE COVID-19 OUTCOMES

Serious outcomes are rare among the children aged 5–11 years. New studies emerged during 2022–24 on the course of COVID-19 infection in infants and the risk factors associated with critical care requirements and deaths in the hospital. Compared with children aged 1–4 years, infants (aged <1 year) had an increased odds ratio (OR) of admission to critical care (OR = 1.63) and death (OR = 2.08), while their sex was not associated with either of these two outcomes. Odds of death were also increased among CYP over 10 years (OR of 2.15 among 10–14-year-olds and >14-year-olds). The number of comorbid conditions was also associated with increased odds of both critical care and death in a dose-response manner: in comparison to no comorbid conditions, ORs for critical care admission were 1.49 for one comorbidity, 2.58 for two, and 2.97 for 3 or more comorbidities. Corresponding ORs for death were 2.15, 4.63, and 4.98, respectively. The odds of admission to critical care were increased for all comorbidities apart from asthma and malignancy. Neurological and cardiac comorbidities were associated with the greatest increase in odds of severe disease or death. Obesity increased the odds of severe disease and death independently of other comorbidities. Taken as a whole, these findings suggest that CYP in higher-risk groups should be prioritised for vaccination [10].

More information was gathered during 2022–24 on children hospitalised for COVID-19. Defining the high-risk group for severe disease among CYP is important for guiding hospital admission and prioritisation for vaccination against SARS-CoV-2. Severe disease occurred among 12.0 per 100 000 children overall and was highest among infants, Hispanic children, and non-Hispanic Black children [11]. In a series of 2293 children who were admitted to hospitals, about 30% had a severe form of the disease and 0.5% died during hospitalisation. Among those aged 0–2 years, severe disease was associated with chronic lung disease (adjusted relative risk (aRR) = 2.2), neurological disorders (aRR = 2.0), cardiovascular disease (aRR = 1.7), prematurity (aRR = 1.6), and airway abnormality (aRR = 1.6). Among children aged 2–17 years who were hospitalised for any reason, the risk of severe COVID-19 was increased among CYP with feeding tube dependence (aRR = 2.0), diabetes mellitus (aRR = 1.9), and obesity (aRR = 1.2) [11].

Another review of 17 articles and a meta-analysis of 10 articles identified significant risk factors for severe COVID-19 in children: neonates (risk ratio (RR) = 2.69), prematurity in young infants (RR = 2.00), obesity (RR = 1.43), diabetes (RR = 2.26), chronic lung disease (RR = 2.62), heart disease (RR = 1.82), neurologic disease (RR = 1.18), immunocompromised status (RR = 1.44), and asthma (RR = 1.08). In the subgroup analysis, being aged younger than three months (RR = 0.26) and having neurodevelopmental disorders (RR = 0.88) were not risk factors for severe COVID-19 [12].

Hospitalisations of children were studied across the USA, particularly for the 5–11-year-old age group with laboratory-confirmed COVID-19. One analysis used the COVID-19-Associated Hospitalization Surveillance Network (COVID-NET) database, which contained data on 1475 children from 14 US states collected between March 2020 and February 2022. Among 397 children hospitalised during the Omicron-predominant period, 87% were unvaccinated, 30% had no underlying medical conditions, and 19% were admitted to an intensive care unit (ICU). The cumulative hospitalisation rate during the Omicron-predominant period was 2.1 times higher among unvaccinated children (19.1 per 100 000 population) than among vaccinated children (9.2 per 100 000 population). Non-Hispanic Black children accounted for the largest proportion of unvaccinated children (34%) and represented approximately one-third of COVID-19-associated hospitalisations in this age group. Children with diabetes and obesity were more likely to experience more severe episodes of COVID-19 [13].

A multicentre, observational cohort analysis from a large regional healthcare system in the Detroit metropolitan area was conducted among paediatric patients to determine if severe COVID-19 outcomes have changed over the course of the pandemic. The authors compared three time intervals: 1 January 2021 to 30 June 2021 for Alpha (T1), 1 July 2021 to 31 December 2021 for Delta (T2), and 1 January 2022 to 16 June 2022 for Omicron variants (T3). The primary outcome was severe disease inclusive of composite ICU admission, mechanical ventilation, multisystem inflammatory syndrome in children (MIS-C), myocarditis, or death. Throughout the pandemic, the proportion of hospitalised infants increased from 16.7% in T1 to 19.6% in T2 to 28.5% in T3. Furthermore, the proportion of teenagers hospitalised decreased from 39.1% in T1 to 31.3% in T2 to 22.1% in T3. Oxygen therapy was required in a minority (29.9%) of cases with supplemental oxygen utilised the least in T3 (16.5%) and most in T2 (30.2%). Composite severe disease decreased throughout the pandemic, occurring in 36.2% of patients in T1, 27.4% in T2, and 18.9% in T3 [14].

Fully vaccinated or fully vaccinated and boosted admission rates remained low throughout all periods, at 4.4% in T1, 4.5% in T2 and 8.4% in T3. Coinfection between SARS-CoV-2 and other viruses was most common during T2 (16.8%) followed by T3 (12.5%), and the least common in T1 (5.1%), while severe outcomes occurred in 45.6% of coinfection cases compared to 22.1% without coinfection [14]. Morphologic and functional low-field-strength magnetic resonance imaging (MRI) can help to identify persistent pulmonary manifestations after SARS-CoV-2 infection. Low-field-strength MRI showed persistent pulmonary dysfunction in children and adolescents who recovered from COVID-19 and those with long COVID [15].

## MORTALITY FROM COVID-19 IN CHILDREN AND YOUNG PEOPLE

Crude COVID-19-related death rates per 1000 person-years for all ages decreased from 4.48 deaths in wave one to 2.69 in wave two, 0.64 in wave three, 1.01 in wave four, and 0.67 in wave five [16]. COVID-19 was the underlying cause of death for at least 1289 CYP aged 0–19 years in the US, with at least 821 CYP deaths occurring in the one-year period from 1 August 2021 to 31 July 2022. This resulted in crude death rates of 1.0 per 100 000 population aged 0–19 years,  4.3 for those <1 year old, 0.6 per 100 000 for 1–4 years old, 0.4 for 5–9 years old, 0.5 for 10–14 years old, and 1.8 for those 15–19 years old. COVID-19 mortality from 1 August 2021 to 31 July 2022 was among the 10 leading causes of death in CYP aged 0 to 19 years in the US, ranking eighth among all causes of death, fifth in disease-related causes of death, and first in deaths caused by infectious or respiratory diseases. COVID-19 deaths constituted 2% of all causes of death in this age group. COVID-19 caused substantially more deaths in CYP annually than any vaccine-preventable disease historically in the recent period before vaccines became available [17].

During the first pandemic year, 3105 of about 12 million CYP living in England died, including 61 who were positive for SARS-CoV-2. Of these deaths, 25 were due to SARS-CoV-2 infection, suggesting a mortality rate of about two per million. Among them, 22 deaths were due to the clinical disease associated with SARS-CoV-2 infection, while three were due to paediatric inflammatory multisystem syndrome, which was temporally associated with SARS-CoV-2. In comparison to all other CYP deaths, CYP older than 10 years, those of Asian and Black ethnic backgrounds, and those with comorbidities were over-represented in SARS-CoV-2-related deaths [18].

## EFFECTS OF ASTHMA ON COVID-19 OUTCOMES

While little is known about the effects of asthma on COVID-19 outcomes in children younger than 12 years, among 35 202 533 adults and 2 996 503 children aged 12–17 years, the risk of death involving COVID-19 for adults with asthma prescribed low-dose inhaled corticosteroids (ICS) was not significantly different from those without asthma after controlling for several potential confounders. Most knowledge on this question comes from the studies of adults with asthma. When prescribed medium and high dosage ICS, adults had an elevated risk of COVID-19 death, with risk increases of 1.18 and 1.36, respectively. For COVID-19 hospitalisation, fully adjusted hazard ratios (HRs) were 1.53 and 1.52 for adults with asthma prescribed medium and high-dosage ICS, respectively. The risk of hospitalisation was greater for children with asthma who had been prescribed one (HR = 2.58) or two or more (HR = 3.80) courses of oral corticosteroids in the year prior to the pandemic. It appears that people with mild and/or well-controlled asthma are neither at significantly increased risk of hospitalisation, nor more likely to die from COVID-19 than adults without asthma [19].

## EFFECTS ON TYPE 1 DIABETES

An analysis of 30 840 children and adolescents with new-onset type 1 diabetes showed that the observed incidences were significantly higher than the predicted incidences (incidence rate ratios (IRRs) of 1.13 in and 1.20 in 2021). The prevalence of autoantibody-negative diabetes did not change (prevalence rate ratio of 0.91 in 2020 and 1.03 in 2021). The incidence of diabetic ketoacidosis during the pandemic was higher than predicted (IRRs of 1.34 in 2020 and 1.37 in 2021). An increase in the incidences of type 1 diabetes and diabetic ketoacidosis, but not of autoantibody-negative diabetes, was observed during both study years [20].

## MULTISYSTEM INFLAMMATORY SYNDROME IN CHILDREN

MIS-C is a severe postinfectious hyperinflammatory condition, which generally occurs 2–6 weeks after a typically mild or asymptomatic infection with SARS-CoV-2. New insights related to MIS-C syndrome were made in 2022–24. A Brighton Collaboration Case Definition of the term ‘Multisystem Inflammatory Syndrome in Children and Adults (MIS-C/A)’ was developed and proposed by topic experts convened by the Coalition for Epidemic Preparedness Innovations for the use in evaluating adverse events following immunisation [21].

Promising new insights from basic research suggested that the transient expansion of TRBV11–2 T cell clonotypes in MIS-C was associated with signatures of inflammation and T cell activation. Also, the association of MIS-C with the combination of HLA A*02, B*35 and C*04 alleles suggests genetic susceptibility. MIS-C B cells showed a higher mutation load than pCOVID-19 and pHC. These results should help in identifying some distinct immunopathological signatures in pCOVID-19 and MIS-C that might help both better diagnosis and treatment [22].

A study of the MIS-C in the US from February 2020 to July 2021 identified 4901 reported cases, with 4470 included in the study. The median patient age was 9 years (interquartile range (IQR) = 5–13) during the third wave, with male predominance (62%) and a highly significant increase in severe haematological and gastrointestinal involvement during the study period. The frequencies of several cardiovascular complications, such as cardiac dysfunction, myocarditis, and shock/vasopressor receipt, as well as renal failure, declined over the study period. Provision of critical care including mechanical ventilation and extracorporeal membrane oxygenation decreased, as did the duration of hospitalisation and mortality [23].

In another USA-based study, surveillance results from 14 December 2020 to 31 August 2021 identified 21 cases of MIS-C after COVID-19 vaccination. The median age was 16 years (range: 12–20), 13 were male and 8 were female; all were hospitalised, with 12 admitted to an ICU; 15 had laboratory evidence of past or recent SARS-CoV-2 infection, and 6 did not. As of 31 August 2021, 21 335 331 individuals aged 12–20 years had received one or more doses of a COVID-19 vaccine. This implies an estimate of the overall MIS-C occurrence after vaccination of 1.0 cases per million individuals receiving one or more doses in this age group, and 0.3 among those without evidence of SARS-CoV-2 infection [24].

In a multicentre study of hospitalised vaccine-eligible US patients aged 5–18 years comparing 304 MIS-C patients with 502 SARS-CoV-2-negative controls, MIS-C was associated with decreased likelihood of vaccination (aOR = 0.16), including among children aged 5–11 years (aOR = 0.22) and 12–18 years (aOR = 0.10), and during the Delta (aOR = 0.06) and Omicron (aOR = 0.22) variant-predominant periods. This association persisted beyond 120 days after the second dose (aOR = 0.08) in 12–18-year-olds. Among all MIS-C case patients, 187 (62%) required ICU admission and 280 (92%) vaccine-eligible case patients were unvaccinated. The study concluded that vaccination with 2 doses of BNT162b2 was associated with a reduced likelihood of MIS-C in children ages 5–18 years [25].

## LONG COVID, POST-ACUTE SEQUELAE OF SARS-COV-2 INFECTION AND POST-COVID CONDITIONS

At least 65 million people worldwide are estimated to have long COVID, with more than 200 symptoms identified with impacts on multiple organ systems [26]. Several reviews of long-term consequences found conflicting reports and an unclear picture of this condition. A review of 14 studies showed that all the studies have major limitations, including the lack of a clear case definition, variable follow-up times, inclusion of children without confirmation of SARS-CoV-2 infection, reliance on self- or parent-reported symptoms without clinical assessment, nonresponse, and other biases, and the absence of a control group [27]. In another review of 17 studies, the pooled prevalence of symptoms in post-COVID participants ranged from 15% (diarrhoea) to 47% (fatigue) [28]. However, later reviews showed that there is a very high variability in terms of the prevalence of various post-acute conditions, ranging from 1.6% to 70.0%. The most frequently reported symptoms were fatigue (2–87%), headache (3–80%), arthro-myalgias (5–66%), chest tightness or pain (1–51%), and dyspnoea (2–57%). Five studies reported limitations in daily function among the analysed populations due to long COVID. Most authors did not report evidence of long-term pulmonary sequelae. Persistent symptoms seemed more common in older age, female sex, and previous long-term pathological conditions among CYP [28,29].

Early reviews agreed that the evidence in CYP on long COVID is limited, heterogeneous, and based on low-quality studies. Progress in understanding should be expected when an agreed definition of long COVID is used, controlled clinical studies are conducted, and the impact of new variants on long COVID prevalence is studied [28,29]. To fully understand long COVID, well-designed prospective studies with representative samples will be essential [30]. In most children, long COVID symptoms resolved within 1–5 months [31].

Another review of 21 studies with a total of 80 071 children and adolescents estimated the prevalence of long COVID to be 25.2%, with the most prevalent clinical manifestations being mood symptoms (16.5%), fatigue (9.7%), and sleep disorders (8.4%). However, some of the numerous limitations of the studies included a lack of standardised definitions, recall, selection, misclassification, nonresponse and/or loss of follow-up, and a high level of heterogeneity [32]. Another review described post-acute complications as persistent symptoms from acute infection (*e.g.* cough, headaches, fatigue, and loss of taste and smell), new symptoms like dizziness, or exacerbation of underlying conditions. It appears that children may develop conditions *de novo*, including postural orthostatic tachycardia syndrome, myalgic encephalomyelitis/chronic fatigue syndrome, autoimmune conditions and multisystem inflammatory syndrome in children [33]. Another review based on 40 studies with 12 424 paediatric cases established the pooled prevalence of any long COVID to be 23.4%. General symptoms were most common (19.6%), followed by respiratory (14.8%), neurologic (13.5%), and psychiatric (12.3%). The most common specific symptoms were dyspnoea (22.8%), fatigue (20.2%), and headache (15.9%). The prevalence of any symptom during 3–6, 6–12, and >12 months was 26.4%, 20.6%, and 14.9%, respectively. Common risk factors were age >10 years, MIS-C, severe clinical symptoms, female sex, poor physical or mental health, or more symptoms [34].

A more recent review of 211 studies covering a population of 13 368 074 individuals suggested that fatigue, dyspnoea, posttraumatic stress disorder, anxiety, and depression were the most frequently reported persistent symptoms after COVID-19. This review confirmed that a higher prevalence of certain symptoms is associated with a more severe illness in the acute phase, European descent, female sex, advanced age, multiple comorbidities, an extended duration of hospital stay, and a high body mass index [35].

Another review suggested the term post-acute sequelae of SARS-CoV-2 infection (PASC) as a synonym for long COVID, describing it as a ‘plethora of unspecific symptoms present later than 4 weeks after confirmed or probable infection with SARS-CoV-2, without another medical explanation’. Furthermore, the term post-COVID conditions (PCC) in children and adolescents is defined by the WHO as ‘PASC occurring within 3 months of acute COVID-19, lasting at least 2 months, and limiting daily activities’. Paediatric PASC mostly remits after a few months, although symptoms can last for more than a year and lead to disability. Fatigue, exertion intolerance, and anxiety are frequent symptoms, while postural tachycardia syndrome and myalgic encephalomyelitis/chronic fatigue syndrome may also occur. There are no diagnostic markers yet, with differential diagnosis being challenging and therapeutic approaches being limited [36].

Specific studies added further interesting insights. In a study conducted across nine children’s hospitals in the USA, the incidence of at least one systemic, syndromic, or medication feature of PASC was 42% among viral test-positive children *vs*. 38% among viral test-negative children. PASC was associated with care in the ICU during the acute illness phase, children younger than five years, and individuals with complex chronic conditions. Myocarditis was the most commonly diagnosed PASC-associated condition [37]. A nationwide cross-sectional study in Denmark showed that, among children aged 0–14 years, cases of COVID-19 had higher odds of reporting at least one symptom lasting more than two months than did controls in the 0–3 years age group (OR = 1.78), 4–11 years age group (OR = 1.23), and 12–14 years age group (OR = 1.21) [38]. In Taiwan, efforts to provide a top-down, nationwide care framework for long COVID patients identified dyspnoea, chronic cough, and fatigue as the most commonly reported symptoms in the first six months after infection, while cognitive impairment and psychological symptoms may persist beyond this time. These symptoms negatively impact individuals' functioning, activities, participation, and quality of life, with rehabilitation being a key element of management to achieving functional improvement [39].

A study conducted by the International Severe Acute Respiratory and Emerging Infection Consortium showed that 126 (24.3%) CYP reported persistent symptoms, among which fatigue (10.7%), sleep disturbance (6.9%), and sensory problems (5.6%) were the most common. Multiple symptoms were experienced by 8.4% of participants. Risk factors for persistent symptoms were older age, defined as 6–11 years (OR = 2.74) and 12–18 years (OR = 2.68), and a history of allergic diseases (OR = 1.67) [40]. Another study in Italy reported that 294 of 1243 CYP had PCC at three months post-onset, 143 patients remained symptomatic at six months, 38 at 12 months, and 15 at 18 months. The following risk factors were associated with PCC: >10 years of age (OR = 1.23), comorbidities (OR = 1.68), and hospitalisation during the acute phase (OR = 4.80). Compared to the Omicron variant, all other variants were significantly associated with PCC at three and six months. At least one dose of vaccine was associated with a reduced, but not statistically significant risk of developing PCC. Most children recovered over time [41].

To address the case definition of PASC/long COVID, a WHO-led Delphi process engaged with an international panel of 265 patients, clinicians, researchers, and WHO staff to develop a consensus definition for this condition. Fourteen domains and 45 items were evaluated in two rounds of the Delphi process to create a final consensus definition [42]. For adults, post-COVID-19 condition occurs in individuals with a history of probable or confirmed SARS-CoV-2 infection, usually three months from the onset, with symptoms that last for at least two months and cannot be explained by an alternative diagnosis. Common symptoms include, but are not limited to, fatigue, shortness of breath, and cognitive dysfunction, and those that generally have an impact on everyday functioning. Symptoms might be new onset following initial recovery from an acute COVID-19 episode or persist from the initial illness. They might also fluctuate or relapse over time. The participating experts agreed that a separate definition might be needed for children [42].

A consensus study was conducted to develop a core outcome set and an associated core outcome measurement set for evaluating post-COVID-19 conditions in CYP. In phase 1, 214 participants from 37 countries participated, with 154 (72%) contributing to both Delphi rounds. The subsequent online consensus meeting resulted in a final core outcome set which encompassed seven critical outcomes: fatigue; post-exertion symptoms; work/occupational changes and study changes; and functional changes, symptoms, and conditions relating to cardiovascular, neuro-cognitive, gastrointestinal and physical outcomes. Four instruments met *a priori* consensus criteria for inclusion: PedsQL multidimensional fatigue scale for ‘fatigue’; PedsQL gastrointestinal symptom scales for ‘gastrointestinal’ outcomes; PedsQL cognitive functioning scale for ‘neurocognitive’ outcomes and EQ-5D for ‘physical functioning’ [43].

Some studies suggest that as few as 1–3% of children recognised with SARS-CoV-2 infection may develop PCC. On its possible pathogenesis, they note that there is increasing evidence about possible abnormalities in the immune responses, cellular metabolism and intestinal microbiota, along with chronic endothelitis. Management of PCC in children requires a multidisciplinary approach, with the goal of offering the best care possible to support diagnostics, research, mental health, and access to research projects [44]. Furthermore, very little is known about the long-term consequences of asymptomatic infection caused by SARS-CoV-2. The rare available sources mention the long-term loss of taste or smell, fatigue, and cough, and agree that the risk of developing long-term symptoms in asymptomatic SARS-CoV-2 infected persons is significantly lower than those in symptomatic cases [45].

## NON-PHARMACEUTICAL INTERVENTIONS-RELATED EFFECTS ON RSV AND INFLUENZA INFECTIONS

Globally, the hospitalisation burden of acute lower respiratory infections (ALRI) associated with the respiratory syncytial virus (RSV) in children younger than five years was significantly reduced during the first year of the COVID-19 pandemic. However, a rebound in hospitalisation rates to pre-pandemic rates was observed by March 2022 in the high-income regions, although this could not be confirmed in middle-income regions. This finding might suggest that negative impact of the pandemic on healthcare systems and healthcare access in middle-income regions persisted longer than in high-income regions [46]. Many countries experienced a relative absence of RSV during the time of a typical season, followed by an out-of-season surge upon relaxation of non-pharmaceutical interventions against COVID-19, disrupting traditional RSV disease patterns and assumptions [47]. Among children younger than five years in England, RSV-associated activity was reduced for all RSV indicators during winter 2020–21, but an unprecedented summer surge of RSV then occurred in 2021, followed by lower-than-expected activity in winter 2021–22. The absence of RSV during winter 2020–21 probably resulted in a cohort of young children who do not have natural immunity to RSV, thereby raising the potential for increased RSV incidence, out-of-season activity, and health-service pressures when measures to restrict SARS-CoV-2 transmission were relaxed [48]. Given that COVID-19 and influenza share the same high-risk groups, it is essential that vaccination programmes target both viruses [49].

## VACCINATION

The debate whether children and young people should be vaccinated against COVID-19 remains open, because there is a relatively low overall health risk from acute COVID-19 in this age group, and because vaccines do not seem to protect as well against Omicron infection and their effect wanes quite quickly – although bivalent vaccines and newer formulations are yet to be fully explored. One of the key arguments for vaccinating healthy CYP is to protect them from long-term consequences, reduce community transmission, and avoid school closures. The most studied vaccines in CYP were BNT162b2 (BioNtech/Fosun Pharma/Pfizer), mRNA-1273 (ModernaTx), ChAdOx1 (Oxford/AstraZeneca), NVX-CoV2373 (Novavax), Ad26.COV2.S (Janssen), CoronaVac (Sinovac), BBIBP-CorV (Sinopharm-Beijing) and BBV152 (Bharat Biotech). The emergence of new variants of concern will require a continuing re-evaluation of the risks and benefits [50]. The United States Centers for Disease Control and Prevention (US CDC) and other public health authorities recommend vaccination of children 12 years or older to protect them, but mostly to contribute to the achievement of herd immunity [51].

Important new knowledge has been generated in 2022–24 on vaccinating CYP against SARS-CoV-2. Several key studies on the basic questions of safety and immunogenicity for various types of vaccines were published for different age groups. A study investigating the safety, immunogenicity, and efficacy of two doses of the BNT162b2 vaccine administered 21 days apart in children 5 to 11 years old showed that the vaccine had a favourable safety profile, with no vaccine-related serious adverse events noted. One month after the second dose, the SARS-CoV-2 neutralising titres were entirely comparable to 16-to-25-year-olds, with a ratio of 1.04. The efficacy against COVID-19 at seven days or more after the second dose was 90.7% [52]. A follow-up study in children six months to less than two years of age, and between two and four years of age also showed that a three-dose primary series of 3-mu g BNT162b2 was safe, immunogenic, and efficacious [53].

Three studies with a similar aim using the mRNA-1273 were conducted first in children aged 12–17 years, followed by the children 6–11 years, and finally in children aged 2–5 and 0.5–2 years. Among the adolescents aged between 12–17 years, the mRNA-1273 vaccine had an acceptable safety profile, while the immune response was similar to that in young adults, and the vaccine was efficacious in preventing COVID-19 [54]. For the children aged 6–11 years, the safety record was excellent, with mainly low-grade, transient adverse events, most commonly injection-site pain, headache, and fatigue; importantly, no vaccine-related serious adverse events, MIS-C, myocarditis, or pericarditis were seen. The neutralising antibody titre was even higher than in young adults, with serologic responses in at least 99.0% of the participants in both age groups. Vaccine efficacy was 88.0% against COVID-19 occurring 14 days or more after the first injection at a time when Delta (B.1.617.2) was the dominant circulating variant [55]. In the follow-up study, children aged 2–5 years had an estimated vaccine efficacy against COVID-19 of 36.8% and those 6-to-23-month-olds had an efficacy of 50.6% at a time when Omicron (B.1.1.529) was the predominant circulating variant. No safety concerns were found in those two age groups, and immune responses were similar, if not better, than those in young adults [56].

The PREVENT-19 trial looked into the safety and immunogenicity of the NVX-CoV2373 in adolescents with a mean age of 13.8 (standard deviation = 1.4) years. Their neutralising antibody geometric mean titres compared with those in young adults were 50% higher. The estimated vaccine efficacy was 79.5% over the entire study period, and was 82.0% specifically for the Delta variant. Reactogenicity was largely mild-to-moderate, with slightly greater frequency after the second dose, while serious adverse events were rare and did not require study discontinuation [57].

The safety, tolerability, and immunogenicity of the CoronaVac vaccine, containing inactivated SARS-CoV-2, in children and adolescents aged 3–17 years was assessed in China. In the combined safety profile, most adverse reactions were mild and moderate, with injection site pain in 2–16% of cases, but CoronaVac was overall well-tolerated and safe. The results supported the use of a two-immunisation schedule for further studies in children and adolescents [58]. A study of an inactivated COVID-19 vaccine, BBIBP-CorV, was also conducted in children aged 3–17 years in China. It showed that the vaccine BBIBP-CorV is safe and well-tolerated, and that it also elicited robust humoral responses against SARS-CoV-2 infection after two doses, supporting a two-shot regimen [59]. The study of recombinant adenovirus type-5 (Ad5)-vectored COVID-19 vaccine with homologous prime-boost regimens in healthy participants aged 6–17 years has shown significant induced RBD-specific ELISA antibodies that decreased with increasing age. A single dose in children and adolescents induced higher antibody responses than that elicited by two doses in adults. The authors concluded that a single dose of Ad5-vectored COVID-19 vaccine is safe, well-tolerated, and induces robust immune responses in children and adolescents aged 6–17 years, but has a limited boosting effect [60].

Two large reviews were helpful in assessing the overall findings that have emerged from numerous vaccine trials on safety and efficacy and were relevant to children and young people. The first one reviewed the information specifically for the 5–11-year-old age group, citing two randomised clinical trials (RCTs) and 15 observational studies involving 10 935 541 vaccinated and 2 635 251 unvaccinated children. It showed that a two-dose mRNA COVID-19 vaccination compared with no vaccination was associated with lower risks of SARS-CoV-2 infections with or without symptoms (OR = 0.47), symptomatic SARS-CoV-2 infections (OR = 0.53), hospitalisations (OR = 0.32), and MIS-C (OR = 0.05). Two RCTs and five observational studies investigated possible vaccine-related adverse effects (AE) among vaccinated children. Most vaccinated children experienced at least one local AE following the first injection and the second injection (86.3%). Vaccination was associated with a higher risk of any AEs compared with placebo (OR = 1.92). The incidence of AEs that prevented normal daily activities was 8.8% and that of myocarditis was estimated to be 1.8 per million following the second injection. The review showed that severe AEs were rare, and that most of these resolved within several days [61].

A large review looked into the efficacy and safety of COVID-19 vaccines in all age groups, including CYP. It included RCTs comparing COVID-19 vaccines to placebo, no vaccine, other active vaccines, or other vaccine schedules; it included 41 RCTs assessing 12 different vaccines, including homologous and heterologous vaccine schedules and the effect of booster doses. The studies on the effect on all-cause mortality were rare, so this effect was convincingly demonstrated only for AD26.COV2.S, with a RR of 0.25 in one large RCT. Then, the reduction in incidence of symptomatic COVID-19 compared to placebo was 97.8% for BNT162b2, 93.2% for mRNA-1273, 70.2% for ChAdOx1, 66.9% for Ad26.COV2.S, 78.1% for BBIBP-CorV, and 77.8% for BBV152. Evidence for the other vaccines was of somewhat lesser certainty, but strong effects were also implied for NVX-CoV2373 (82.9%), and CoronaVac (69.8%). The evidence of a reduction in the incidence of severe or critical disease due to COVID-19 compared to placebo was of high certainty for BNT162b2 (95.7%), mRNA-1273 (98.2%), AD26.COV2.S (76.3%), BBV152 (93.4%), of moderate certainty for NVX-CoV2373 (VE 82.9%), and of low certainty for CoronaVac (69.8%). According to this review, the vaccines with no demonstrable serious adverse events in comparison to placebo were mRNA-1273, ChAdOx1, Ad26.COV2.S, and BBV152, whereas the conclusion is similar, but the evidence less certain for BNT162b2, CoronaVac, BBIBP-CorV, and NVX-CoV2373 when compared to placebo [62].

In the USA, following the rise of the Omicron variant, vaccine effectiveness (VE) against laboratory-confirmed COVID-19 leading to hospitalisation and against critical COVID-19 leading to receipt of life support or to death was assessed from 1 July 2021 to 17 February 2022 at 31 hospitals in 23 states. During the Delta-predominant period, VE against hospitalisation for COVID-19 among adolescents 12–18 years was 93% from 2 to 22 weeks after vaccination, and 92% at 23–44 weeks. During the Omicron-predominant period, VE in adolescents 12–18 years was 40% against hospitalisation for COVID-19, 79% against critical COVID-19, and 20% against non-critical COVID-19. The study estimated the VE estimates for children aged 5–11 years during the Omicron period, with VE against hospitalisation being 68% [63]. Another study in the USA based on combined data from three prospective cohort studies from six sites noted that bivalent mRNA COVID-19 vaccines were recommended in the US for children and adolescents aged 12 years or older on 1 September 2022, and for children aged 5 to 11 years on 12 October 2022. Between 4 September 2022 and 31 January 2023, VE was assessed among children and adolescents aged 5–17 years based on weekly self-collected nasal swabs. Bivalent VE against SARS-CoV-2 infection was 54.0% and VE against symptomatic COVID-19 was 49.4% (95% CI = 22.2–70.7) [64].

The third notable study from the US, based on the PROTECT cohort study monitoring SARS-CoV-2 infections among participants aged 0.5–17 years in Arizona, Florida, Texas, and Utah, initiated in July 2021, studied the effectiveness of two-dose BNT162b2 in preventing SARS-CoV-2 infection among children aged 5–11 years and adolescents aged 12–15 years. Approximately one half (51%) of all Omicron infections were asymptomatic compared with approximately one third (34%) of Delta infections. Among 5–11-year-olds, adjusted VE against symptomatic and asymptomatic Omicron infection 14–82 days after receipt of dose 2 was 31%. Among the adolescents aged 12–15 years, the adjusted VE at 14–149 days after receipt of dose 2 was 87% against Delta and 59% against Omicron [65].

A fourth study from the US used data from 6897 pharmacy-based, drive-through SARS-CoV-2 testing sites from a single pharmacy chain and included 74 208 tests from children aged 5–11 years and 47 744 tests from adolescents aged 12–15 years between 26 December 2021 and 21 February 2022. At two to four weeks after the second dose, the estimated VE against symptomatic infection among children 5–11 years was 60.1%, while that among adolescents aged 12–15 years was 59.5%. During the second month after the second dose, VE decreased to 28.9% among children and 16.6% among adolescents. Among the latter group, the booster dose increased VE to 71.1%. The authors concluded that, among children and adolescents, the estimated VE for two doses of BNT162b2 against symptomatic infection was modest and decreased rapidly, but among adolescents, the estimated VE increased after a booster dose [66].

In Singapore, the study of the effectiveness of BNT162b2 vaccine against Omicron in 255 936 children aged 5–11 years was conducted from 21 January through 8 April 2022, when the Omicron variant was spreading rapidly. Among unvaccinated children, the crude incidence rates of all reported SARS-CoV-2 infections, PCR-confirmed SARS-CoV-2 infections, and COVID-19-related hospitalisations were 3303.5, 473.8, and 30.0 per one million person-days, respectively. Among partially vaccinated children, VE was 13.6% against all SARS-CoV-2 infections, 24.3% against PCR-confirmed SARS-CoV-2 infection, and 42.3% against COVID-19-related hospitalisation. In fully vaccinated children who had received at least two doses, the respective VE was 36.8%, 65.3%, and 82.7% [67].

In Italy, a retrospective analysis of the effectiveness of BNT162b2 vaccine against SARS-CoV-2 infection and severe COVID-19 in children aged 5–11 years was conducted from January to April 2022. By 13 April, less than 40% of 5–11-year-olds in Italy had been vaccinated against COVID-19. Overall, VE in the fully vaccinated group was 29.4% against SARS-CoV-2 infection and 41.1% against severe COVID-19, while VE in the partly vaccinated group was 27.4% against SARS-CoV-2 infection and 38.1% against severe COVID-19. Analysis of waning showed that VE decreased to 21.2% at 43–84 days after full vaccination [68].

The partnership of the US CDC and the VISION Network examined 39 217 emergency department (ED) and urgent care (UC) encounters and 1699 hospitalisations among persons aged 5–17 years with COVID-19-like illness across 10 states between April 2021 to January 2022 to estimate VE. Among children aged 5–11 years, VE against laboratory-confirmed COVID-19-associated ED and UC encounters 14–67 days after dose 2 was 46%, while among adolescents aged 12–15 and 16–17 years, VE at 14–149 days after dose 2 was 83% and 76%, respectively. Among adolescents aged 16–17 years, VE increased to 86% ≥7 days after the third (booster) dose. Moreover, VE against COVID-19-associated ED and UC encounters was substantially lower during the Omicron period than the Delta period among adolescents aged 12–17 years. Overall, two-dose VE against COVID-19-associated hospitalisation was 73–94% [69].

A study conducted in England was particularly useful because it showed how the protection from previous infection compared with protection from vaccination in adolescents aged 12–17 years. It also evaluated the protection of hybrid immunity. In unvaccinated adolescents aged 12–17 years, previous SARS-CoV-2 infection with wildtype, Alpha, or Delta strains provided greater protection against subsequent Delta infection (>86.1%) than against subsequent Omicron infection (<52.4%), while previous Delta or Omicron infection provided similar protection against Omicron reinfection (52.4% *vs*. 59.3%). Furthermore, in adolescents with no previous infection, vaccination provided lower protection against Omicron infection than against Delta infection, with Omicron protection peaking at 64.5% at 2–14 weeks after dose two and 62.9% at 2–14 weeks after dose three, with waning protection after each dose. Adolescents with hybrid immunity from previous infection and vaccination had the highest protection, irrespective of the SARS-CoV-2 strain in the primary infection. The highest protection against Omicron infection was observed in adolescents with vaccination and previous Omicron infection, reaching 96.4% at 15–24 weeks after the second vaccine dose [70].

A systematic review and meta-analysis of the safety and effectiveness of vaccines against COVID-19 in children aged 5–11 years assessed that more than half of all children globally are estimated to be seropositive, with low rates of severe COVID-19. The review was based on the evidence up to 23 January 2023, including studies with participants aged 5–11 years with BNT162b2 (Pfizer-BioNTech), BNT162b2 Bivalent (against original strain and omicron (BA.4 or BA.5)), mRNA-1273 (Moderna), or mRNA-1273.214 (against original strain and Omicron BA.1). Here, VE after two doses was 41.6% against Omicron infections, 36.2% against symptomatic COVID-19, 75.3% against COVID-19-related hospitalisations, and 78.0% against MIS-C. It was not possible to estimate VE against COVID-19-related mortality, but crude event rates for deaths in unvaccinated children were less than one case per 100 000 children, and no events were reported for vaccinated children. After three doses, VE against Omicron infections was 55%, while that against symptomatic COVID-19 it was 61%, with studies reporting vaccine efficacy or effectiveness against hospitalisation following a third dose. Safety data suggested no increased risk of serious adverse events. The review concluded that, in children aged 5–11 years, mRNA vaccines are moderately effective against infections with the Omicron variant, but probably protect well against COVID-19 hospitalisations [71]. Another systematic review and other studies also concluded that protection against Omicron infection is inferior to protection against Delta and Alpha infections and that it wanes faster over time. Booster mRNA vaccines can reestablish effectiveness, although the degree of protection against Omicron is only partial [72].

## VACCINE PROTECTION AGAINST MIS-C AND LONG COVID

In the USA, among 102 MIS-C case patients and 181 hospitalised controls, the estimated VE of two doses of BNT162b2 (Pfizer-BioNTech) vaccine against MIS-C was 91%. All 38 MIS-C patients requiring life support were unvaccinated. Receipt of two doses of the Pfizer-BioNTech vaccine was associated with a high level of protection against MIS-C in persons aged 12–18 years, highlighting the importance of vaccination among all eligible children [73]. Further, VE against long COVID in children aged 5–17 years was also estimated through a retrospective cohort study using data from 17 health systems in the RECOVER PCORnet electronic health record programme for visits after vaccine availability. In the cohort of 1 037 936 children, 67% were vaccinated. Among patients with COVID-19, the incidence of probable long COVID was 4.5%, and that of diagnosed long COVID was 0.8%. Adjusted VE within 12 months was 35.4% against probable long COVID and 41.7% against diagnosed long COVID. VE was higher for adolescents (50.3%) than younger children, and it was higher at 6 months (61.4%) and then decreased to 10.6% at 18 months [74].

## MYOCARDITIS AND PERICARDITIS AS VACCINE SAFETY ISSUES

Several studies tried to shed light on the question of myocarditis and pericarditis, both as possible serious AEs of vaccines and as a possible complication of COVID-19 among CYP. Passive surveillance reporting in the USA assessed the risk of myocarditis after receiving mRNA-based COVID-19 vaccines across multiple age and sex strata. Among 1991 reports of myocarditis to the Vaccine Adverse Effects Reporting System (VAERS), 1626 met the case definition of myocarditis, with a median age of 21 years (IQR = 16–31). The median time to symptom onset was 2 days (IQR = 1–3). Males comprised 82% of the myocarditis cases, and the rates were highest after the second vaccination dose in adolescent males aged 12–15 years (7.1 per 100 000 doses of the BNT162b2 vaccine), in adolescent males aged 16–17 years (10.6 per 100 000 doses of the BNT162b2 vaccine), and in young men aged 18–24 years (5.2 and 5.6 per 100 000 doses of the BNT162b2 vaccine and the mRNA-1273 vaccine, respectively). There were 826 cases of myocarditis among those younger than 30 years. Among them, 98% had elevated troponin levels, 72% had abnormal electrocardiogram results, and 72% had abnormal cardiac magnetic resonance imaging results. Approximately 96% were hospitalised and 87% had resolution of presenting symptoms by hospital discharge. The most common treatment was nonsteroidal anti-inflammatory drugs, which were administered in 87% of cases [75].

A population-based cohort study of Ontario residents following mRNA COVID-19 vaccination included 19 740 741 doses of mRNA vaccines. Among them, there were 297 reports of myocarditis or pericarditis, 228 (76.8%) occurring in males, with the median age of 24 years (range: 12–81 years), and 207 (69.7%) occurring after the second dose of the COVID-19 mRNA vaccine. Males aged 18–24 years following the second dose of mRNA-1273 had an incidence of 30.0 cases per 100 000 doses. This rate was lower following the second dose of BNT162b2, at 5.9 cases per 100 000 doses. Overall rates for both vaccine products were significantly higher when the inter-dose interval was 30 or fewer days compared with 56 or more days [76].

In England, a self-controlled case series study of people aged ≥13 years showed that myocarditis occurred in 2861 (0.007%) people, with 617 events occurring 1–28 days after vaccination. The risk of myocarditis was increased in the 1–28 days after the first dose of ChAdOx1 (IRR = 1.33) and a first, second, and booster dose of BNT162b2 (IRRs of 1.52, 1.57, and 1.72, respectively), but was lower than the risks after a positive SARS-CoV-2 test before or after vaccination (IRRs of 11.14 and 5.97, respectively). The risk of myocarditis was higher 1–28 days after a second dose of mRNA-1273 (IRR = 11.76) and persisted after a booster dose (IRR = 2.64). Associations were stronger in men younger than 40 years for all vaccines. In men younger than 40 years old, the number of excess myocarditis events per million people was higher after a 2nd dose of mRNA-1273 than after a positive SARS-CoV-2 test (97 *vs*. 16). In women younger than 40 years, the number of excess events per million was similar after a second dose of mRNA-1273 and a positive test (7 *vs*. 8). Overall, the risk of myocarditis is greater after SARS-CoV-2 infection than after COVID-19 vaccination [77].

Another self-controlled case series in England using individual-patient-level data from over 38 million people aged ≥16 years found an increased risk of myocarditis within a week of receiving a first dose of ChAdOx1, BNT162b2, and mRNA-1273 vaccines, which was further increased after a second dose of either mRNA vaccine. SARS-CoV-2 infection was associated with an even greater risk of myocarditis, as well as pericarditis and cardiac arrhythmia. The study estimated an extra two, one, and six myocarditis events per one million people vaccinated with ChAdOx1, BNT162b2 and mRNA-1273, respectively, in the 28 days following a first dose. After the second dose, this increased to 10 myocarditis events per one million vaccinated in the 28 days after a second dose of mRNA-1273. This compares with an extra 40 myocarditis events per one million patients in the 28 days following a SARS-CoV-2 positive test. Risks of pericarditis and cardiac arrhythmias were also increased following a positive SARS-CoV-2 test [78].

In Denmark, Finland, Norway, and Sweden, a study evaluating the risk of myocarditis in 12–39-year-olds following a booster dose of a COVID-19 vaccine was based on nationwide registers and included 8.9 million individuals. It identified 1533 cases of myocarditis. The 28-day acute risk period following the third dose of BNT162b2 or mRNA-1273 was associated with an increased incidence rate of myocarditis compared to the post-acute risk period 28 days or more after the second dose, with IRRs of 2.08 and 8.89, respectively. For females, the corresponding IRR was only estimable for BNT162b2 and was found to be 3.99. The corresponding absolute risks following the third dose of BNT162b2 and mRNA-1273 in males were 0.86 and 1.95 myocarditis events within 28 days per 100 000 individuals vaccinated, respectively. In females, the corresponding absolute risk following the 3rd dose of BNT162b2 was 0.15, with no deaths occurring within 30 days of vaccine-related cases [79].

A systematic review and meta-analysis based on 22 studies and 405.3 million vaccine doses assessed the overall incidence of myopericarditis at 33.3 cases (95% confidence interval = 15.3–72.6) per million vaccine doses. Compared with COVID-19 vaccination, the incidence of myopericarditis was significantly higher following smallpox vaccinations (132.1; *P* < 0.0001), but was not significantly different after influenza vaccinations (1.3; *P* = 0.43) or in studies reporting on various other non-smallpox vaccinations (57.0; *P* = 0.58). Among people who received COVID-19 vaccines, the incidence of myopericarditis was significantly higher in males, in people younger than 30 years, after receiving an mRNA vaccine, and after a second dose of vaccine, as previously noted in a number of national studies [80].

An extensive meta-analysis of 11 studies with 58 620 611 subjects confirmed that COVID-19 vaccination correlated with an increased risk of myocarditis or pericarditis (RR = 2.04). In addition, an increased risk of myocarditis or pericarditis in people who received the second dose of the COVID-19 vaccine compared with that in those who received only the first dose of the COVID-19 vaccine was also found (RR = 4.06). An increased incidence of pericarditis or myocarditis was noted predominantly in those who received BNT162b2 and mRNA-1273 vaccines (RRs of 2.19 and 4.15, respectively). Still, the risks of myocarditis and pericarditis in COVID-19 vaccine recipients are still significantly lower than the health risks observed in patients with COVID-19 [81].

After the US CDC's October 2022 recommendation for bivalent COVID-19 booster vaccination for children aged 5–11 years in the US, most VAERS reports represented vaccine errors rather than adverse events. Neither myocarditis nor death were reported after bivalent booster vaccination [82]. In England, a self-controlled case-series study was conducted using linked data of 5.1 million children to study COVID-19 vaccine safety. In 5–11-year-olds, there were no increased risks of adverse events 1–42 days following vaccination with BNT162b2, mRNA-1273, or ChAdOX1. In 12–17-year-olds, there were an estimated 3 and 5 additional cases of myocarditis per million following a first and second dose with BNT162b2, respectively. An additional 12 hospitalisations with epilepsy and 4 with demyelinating disease (in females only, mainly optic neuritis) were estimated per million following a second dose of BNT162b2 [83].

In conclusion, the risk of acute myocarditis associated with COVID-19 mRNA vaccination has garnered intense (social) media attention. However, myocarditis after COVID-19 mRNA vaccination is rare and usually resolves within days or weeks. Moreover, the risks of hospitalisation and death associated with COVID-19 are greater than the risks associated with COVID-19 vaccination. Therefore, COVID-19 vaccination should be recommended in adolescents and adults [84].

## VACCINE HESITANCY

A systematic review of 98 studies across 69 different countries with 413 590 participants estimated the prevalence of parental acceptance to vaccinate their children with the COVID-19 vaccine to be about 57%. Parents' COVID-19 vaccine knowledge, trust in the COVID-19 vaccine, and facilitators in vaccination (*e.g.* low cost, good vaccine accessibility, and government incentive) were significant factors for higher willingness, while mental health problems (*e.g.* having worries and psychological distress) were significant factors for lower willingness [85]. Another study among 23 000 respondents in 23 countries surveyed from June to July 2022 found willingness to accept vaccination at 79.1%, up by 5.2% from June 2021. An increase in hesitancy over the same period was noted in eight countries, by as little as 1.0% in the United Kingdom to 21.1% in South Africa. About 12% vaccinated respondents are hesitant about booster doses. Overall support for vaccinating children under 18 years of age increased slightly. Almost two in five (38.6%) respondents reported paying less attention to new COVID-19 information than previously [86].

In the USA, from November to December 2022, only 27.1% of adults and 18.5% of adolescents who had completed a COVID-19 primary series received a bivalent booster, and coverage was lower among Black and Hispanic persons. An additional 39.4% of adults were open to booster vaccination, and an additional 52.0% of adolescents had parents who were open to booster vaccination for their children. Those in rural areas had much lower primary series completion rate and up-to-date vaccination coverage. Healthcare provider recommendations for booster vaccination, dissemination of information about the safety of vaccine by trusted messengers, and reducing barriers to vaccination could all improve COVID-19 booster vaccination coverage [87]. In Austrian adolescents, willingness to receive COVID-19 vaccination was higher in students compared to apprentices, and migration background and female gender were associated with lower vaccination willingness [88].

## ETHNIC DIFFERENCES IN VACCINE COVERAGE

The study in the USA looked at the ethnic differences in COVID-19 vaccination coverage among CYP aged 5–17 years from December 2020 to September 2022. The US CDC analysed data from the National Immunization Survey-Child COVID Module. By 31 August 2022, approximately one-third (33.2%) of children aged 5–11 years, more than one-half (59.0%) of children and adolescents aged 12–15 years, and more than two-thirds (68.6%) of adolescents aged 16–17 years had received ≥1 COVID-19 vaccine dose. Vaccination coverage was highest among non-Hispanic Asian children and adolescents, ranging from 63.4% among those aged 5–11 years to 91.8% among those aged 16–17 years. Coverage was next highest among Hispanic or Latino (Hispanic) children and adolescents (34.5%-77.3%). Coverage was similar for non-Hispanic Black or African American, non-Hispanic White, and non-Hispanic other ethnicity or multiple ethnicity CYP. Among children aged 5–11 years, coverage among Black children was lower than that among Hispanic, Asian, and other/multiple ethnicity children [89].

## EFFECTS OF UNDER-VACCINATION

The proportion of under-vaccinated people, *i.e. *those who received fewer than the recommended number of doses, was 45.8% in England, 49.8% in Northern Ireland, 34.2% in Scotland, and 32.8% in Wales on 1 June 2022. The potential for reduction in severe COVID-19 outcomes in the UK over 4 months of follow-up associated with a counterfactual scenario in which everyone was fully vaccinated on 1 June 2022 was only 210 cases in the 5–15 years age group [90].

## EFFECTS ON MENTAL HEALTH AND EDUCATION

COVID-19 is associated with increased risks of neurological and psychiatric sequelae in the weeks and months thereafter. An analysis of two-year retrospective cohort studies of individuals diagnosed with COVID-19 showed that the increased incidence of mood and anxiety disorders was transient, with no overall excess of these diagnoses compared with other respiratory infections. In contrast, the increased risk of psychotic disorder, cognitive deficit, dementia, and epilepsy or seizures persisted throughout. The differing trajectories between the two sets of outcomes suggest a different pathogenesis. Children have a more benign overall profile of psychiatric risk than adults and older adults, but their sustained higher risk of some diagnoses is of concern [91]. In another study, adolescents and young adults at an early epicentre of the COVID-19 pandemic in the US experienced increased depression and anxiety symptoms, particularly amongst females. School and home confinement concerns related to the pandemic were independently associated with changes in symptoms [92].

An increased prevalence of COVID-19-related fear is sometimes mentioned in relation to CYP. However, coping strategies exist, such as physical exercise, access to entertainment, positive familial relationships, and social support were associated with better mental health outcomes [93]. A parental survey during the second German school lockdown showed that children's learning time decreased during the first school closures, particularly for low-achieving students, and increased only slightly 1 year later. Parental assessments of children's socioemotional development were mixed [94]. In the UK, amidst rapidly changing political and economic circumstances, continuing precarity for young people seems to be one certainty [95]. Another study suggested that child problem behaviours and emotional well-being improved after the first lockdown and during subsequent periods of relaxation before worsening again in the second lockdown. Importantly, parental stress emerged as a strong risk factor, and the parent-child relationship quality constituted a resilience factor for children’s psychological well-being, although the noted associations were weak and difficult to study [96].

## REMAINING QUESTIONS FOR FUTURE RESEARCH

Several important questions remain unanswered. The long-term consequences of SARS-CoV-2 infections, including the potential for lingering pulmonary dysfunction and its broader impact on childhood development, are not yet fully understood. The mechanisms driving the increased incidence of type 1 diabetes following COVID-19, as well as the genetic and immunological factors underlying susceptibility to severe outcomes such as MIS-C, require further investigation. Additionally, while vaccination appears to reduce the likelihood of severe disease and hospitalisation, the durability of immunity – both from natural infection and vaccination – remains an area of ongoing research. As new variants continue to emerge, continuous surveillance and adaptive public health policies will be crucial to safeguarding paediatric populations against both acute and long-term COVID-19 complications.

Further research is expected on the pathophysiology of long COVID in children, including the role of immune dysregulation, metabolic abnormalities, and microbiome alterations. Vaccination also appears to reduce the risk of long COVID, although the exact mechanisms of protection and the durability of this effect are still not fully understood. The long-term consequences of SARS-CoV-2 infection, particularly among asymptomatic cases, are still unclear, and little is known about how different variants or hybrid immunity affect the risk of PCC. Additionally, the relative efficacy of different therapeutic interventions, including rehabilitation and mental health support, also requires further investigation. The impact of COVID-19 on the development of chronic conditions such as myalgic encephalomyelitis/chronic fatigue syndrome and postural orthostatic tachycardia syndrome in children remains another area to study. As societies continue to navigate COVID-19’s aftermath, policies ensuring equitable vaccine distribution, effective booster strategies, and targeted interventions to mitigate the pandemic’s broader impacts on young populations will be of interest.

## CONCLUSIONS

The research conducted between 2022 and 2024 has advanced our understanding of COVID-19 in children and young people, particularly with the emergence of the Omicron variant and its subvariants. The findings reinforce that while Omicron infections are often milder compared to earlier variants, the overall seroprevalence of SARS-CoV-2 in children has increased, with notable regional and demographic disparities. Hospitalisation rates in children surged during Omicron waves, especially among infants, unvaccinated individuals, and those with comorbidities such as obesity, diabetes, and neurological or cardiac conditions. Despite this, severe disease and mortality in children remain rare. The observed increases in type 1 diabetes incidence and MIS-C also highlight the broader systemic effects of SARS-CoV-2 in paediatric populations. Additionally, existing evidence underscores the protective effect of vaccination in preventing severe disease and MIS-C, emphasising the need for targeted immunisation strategies, particularly among high-risk groups.

Studies estimate that a significant proportion of children experience persistent symptoms such as fatigue, mood disturbances, sleep disorders, and respiratory difficulties. However, reported prevalence varies widely, from as low as 1.6% to as high as 70%, due to differences in study methodologies, case definitions, and populations studied. Standardised definitions and measurement tools, such as those developed through international consensus processes, are crucial for improving diagnosis, treatment, and research into this condition. While myocarditis and pericarditis emerged as rare but concerning side effects following mRNA vaccination – most notably in young males – the risk remains significantly lower than that associated with SARS-CoV-2 infection itself. Ethnic disparities in vaccine uptake persist, with lower coverage observed among Black and Hispanic children in the USA, while Asian children exhibit the highest vaccination rates, implying vaccine hesitancy, accessibility and countering disinformation as important areas for future research.
